# Diagnostic evidence in suspected discogenic low back pain: a pain-source attribution perspective

**DOI:** 10.3389/fmed.2026.1924460

**Published:** 2026-08-10

**Authors:** Renpan Zhang, Yaqian Liang, Chenchao Zhang, Yaxin Liu, Youyi Hong

**Affiliations:** Department of Pain Medicine, Quanzhou Orthopedic-Traumatological Hospital, Quanzhou, Fujian, China

**Keywords:** clinical decision-making, diagnostic blocks, discogenic low back pain, magnetic resonance imaging, provocative discography

## Abstract

Discogenic low back pain (DLBP) remains difficult to identify clinically because degenerative disc changes are common and symptom patterns overlap with other lumbar or pelvic pain sources. This mini review summarizes evidence relevant to suspected DLBP from a pain-source attribution perspective, focusing on clinical features, disc and endplate magnetic resonance imaging (MRI) findings, assessment of other pain sources, provocative discography, and diagnostic blocks. Clinical features such as axial low back pain, sitting or flexion intolerance, non-radicular referral, and centralization may increase suspicion of disc-related pain, but they have limited specificity when used alone. MRI can identify high-intensity zones, Modic changes, and other disc- or endplate-related abnormalities that support structural compatibility when findings correspond to the suspected level. Assessment of other pain sources helps refine the differential diagnosis and reduces overinterpretation of disc-related imaging findings. Provocative discography and diagnostic blocks provide procedural information through reproduction of familiar pain and pain change after targeted anesthesia, respectively, but these responses require interpretation alongside clinical and imaging evidence. Overall, suspected DLBP is more strongly supported when clinical presentation, level-concordant imaging findings, assessment of other pain sources, and procedural responses point in the same direction within the same patient. Future studies should evaluate whether combined diagnostic information improves diagnostic agreement and patient selection for further diagnostic evaluation.

## 1 Introduction

Low back pain is a leading cause of disability worldwide and imposes a substantial clinical and socioeconomic burden (1, 2). In the clinical evaluation of patients with chronic low back pain, discogenic low back pain (DLBP) is one potential pain source that may need to be considered (3–6). However, degenerative disc findings on magnetic resonance imaging (MRI) cannot be equated directly with discogenic pain, because similar imaging changes are also observed in asymptomatic individuals (7, 8). Therefore, the evaluation of suspected DLBP should not rely on disc degeneration alone, but should consider these findings in relation to the patient's clinical presentation.

Clinical evaluation of chronic low back pain is also influenced by multiple potential pain sources and overlapping symptom patterns. Similar pain presentations do not necessarily arise from the intervertebral disc and may involve other lumbar or pelvic sources. This overlap can make it difficult to determine whether disc-related findings are clinically relevant to the patient's current pain.

The interpretation of existing evidence is further affected by variation across studies. Definitions of DLBP, patient selection criteria, and reference standards have not been consistent, and studies using the same diagnostic label may have identified patients under different diagnostic conditions. These differences limit direct comparison across studies and influence how individual findings can be applied to the clinical evaluation of suspected DLBP.

Previous reviews have addressed the definitions, mechanisms, imaging findings, and treatment options for discogenic low back pain (3–6). This mini review examines diagnostic evidence relevant to suspected DLBP, including clinical features, disc and endplate MRI findings, assessment of other lumbar or pelvic pain sources, provocative discography, and diagnostic blocks. The aim is to clarify how these findings may inform clinical diagnostic judgment, together with their limitations, in the evaluation of suspected DLBP.

## 2 Review methods

This mini review was designed as a focused clinical review of evidence relevant to the diagnostic evaluation of suspected DLBP. Peer-reviewed literature was identified through PubMed/MEDLINE searches up to 20 May 2026, supplemented by screening the reference lists of relevant diagnostic studies, reviews, consensus documents, and methodological or technical articles.

Eligible literature included studies, reviews, and technical or methodological reports that could inform the clinical evaluation of suspected DLBP. The review focused on clinical features, magnetic resonance imaging (MRI) findings, assessment of other lumbar or pelvic pain sources, provocative discography, discoblock, diagnostic blocks, and the interpretation of diagnostic findings in the absence of a uniform reference standard. Literature focused solely on treatment efficacy, surgical outcomes without diagnostic context, non-lumbar conditions, or unrelated causes of low back pain was not included as core diagnostic evidence.

The literature was organized according to the type of diagnostic evidence addressed. Findings were summarized qualitatively to describe the diagnostic contribution, interpretive limitations, and clinical context of each evidence area. Because the purpose of this review was to provide a focused clinical synthesis rather than a diagnostic-test-accuracy meta-analysis, evidence was not pooled quantitatively.

## 3 Diagnostic evidence for suspected DLBP

### 3.1 Diagnostic evidence domains

For presentation, the evidence is summarized in five areas: clinical features, disc and endplate MRI findings, alternative pain-source assessment, provocative discography, and diagnostic blocks. Table 1 summarizes representative findings and key limitations across these areas. Table 2 summarizes procedural factors and response patterns that affect the interpretation of provocative discography and diagnostic blocks.

**Table 1:** Representative diagnostic evidence in suspected DLBP.

Evidence area	Representative findings	Diagnostic contribution	Interpretive condition
Clinical features	Axial low back pain, sitting or flexion intolerance, non-radicular referral, and centralization (+LR ~2.8–3.1) (3, 9–12).	Defines the clinical context in which disc-related pain is considered and helps identify patients for further diagnostic evaluation.	Best interpreted together with imaging, differential pain-source assessment, and, when appropriate, procedural findings.
Disc and endplate MRI findings	Age-related degeneration is common (8); HIZ specificity ~0.89, +LR ~4.52, and AUC ~0.84 in one meta-analysis (14); lumbar stability, Modic change, and HIZ were reported as independent imaging factors in one retrospective study (18).	Provides disc and endplate structural information, especially when findings match symptoms and the suspected spinal level.	Most informative when abnormality type, symptom pattern, and level concordance are reported.
Alternative pain-source assessment	Facet joint, sacroiliac joint, radicular, hip, and mixed pain-source evaluations (19, 20, 40, 41); SIJ test clusters +LR ~2.4–3.2 (12, 41).	Supports differential diagnosis and helps determine whether disc-related findings remain the leading diagnostic consideration.	Interpretation depends on clinical examination, block criteria, and overlap among lumbar and pelvic pain sources.
Provocative discography	Concordant pain reproduction; false-positive estimates ~9.3% per patient and ~6.0% per disc; pooled specificity ~0.94 (21–25).	Provides controlled provocation evidence through reproduction of familiar pain at a tested disc.	Most informative with pressure or volume control, disc morphology, control-level responses, and clinical/MRI concordance.
Diagnostic blocks	Discoblock, L2 spinal nerve root infiltration, and selective nerve blocks; partial agreement with provocative discography (26–28).	Provides diagnostic information through pain relief or pain modification after targeted anesthesia.	Most informative when response criteria, target selection, and relation to discography or MRI findings are reported.

DLBP, discogenic low back pain; HIZ, high-intensity zone; +LR, positive likelihood ratio; SIJ, sacroiliac joint.

**Table 2:** Procedural findings and interpretive safeguards in suspected DLBP.

Procedural finding or context	Diagnostic information captured	Representative evidence or parameter	Interpretive contribution	Interpretive safeguard
Provocative discography	Reproduction of familiar pain during stimulation of the tested disc.	Concordant pain reproduction interpreted with disc morphology and procedural conditions (21–25).	Supports level-specific disc-source attribution when clinical and MRI findings are concordant.	Interpret positive responses with pressure/volume conditions, disc morphology, and control-level responses.
Pressure/volume context	Pain response recorded under defined stimulation conditions.	Mean intradiscal peak pressure 15.1 psi; adjacent-disc pressure rise absent in 48 procedures and 1.1 psi in 2 procedures (25).	Clarifies the stimulation context for provoked pain responses.	Pressure and volume information should contextualize, not replace, clinical and MRI concordance.
Control-level response	Difference between index-disc and adjacent/control-level responses.	Control-level interpretation used to improve level-specific interpretation (21, 23–25).	Helps judge whether pain reproduction is specific to the suspected level.	Pain at control levels reduces confidence in attributing pain to the index disc alone.
False-positive context	Background risk that positive provocation may occur outside the suspected pain source.	False-positive estimates: 9.3% per patient, 6.0% per disc; pooled specificity 0.94 (21).	Frames discography results within known methodological limitations.	Interpret within patient selection, psychosocial context, procedural technique, and other diagnostic evidence.
Diagnostic blocks	Pain relief or modification after targeted anesthetic intervention.	Discoblock, L2 spinal nerve root infiltration, and selective nerve blocks evaluated symptom modification (26–28).	Provides complementary functional information based on response to targeted anesthesia.	Response criteria, target selection, and relation to discography/MRI findings should be specified.
Cross-procedure agreement	Concordance or discordance between pain provocation and pain relief procedures.	L2 infiltration vs. discography: agreement 46.5%, false-positive 20.5%, false-negative 33% (26).	Helps compare whether different functional responses point to the same suspected source.	Agreement supports converging interpretation; discordance signals that response type and target specificity differ.

Diagnostic blocks included discoblock and selective nerve-related blocks interpreted according to pain reproduction, pain relief, or pain modification.

DLBP, discogenic low back pain; L2, second lumbar spinal nerve; psi, pounds per square inch.

### 3.2 Clinical features

Clinical feature studies described axial low back pain, sitting or flexion intolerance, and non-radicular referred pain in patients with suspected discogenic pain (3, 9, 10). Among clinical findings with diagnostic accuracy estimates, centralization was the most consistently reported. In a systematic review of tests used to identify disc, sacroiliac joint, or facet joint pain sources, centralization increased the probability of a disc source, with a positive likelihood ratio of 2.8 (95% CI, 1.4–5.3) (11). A more recent diagnostic accuracy review reported similar findings, with a pooled positive likelihood ratio of 3.06 (95% CI, 1.44–6.50) and a negative likelihood ratio of 0.66 (95% CI, 0.52–0.84) for centralization (12). A medical-interview study in patients with DLBP associated with degenerative disc disease reported a five-item support tool with an AUC of 0.923, sensitivity of 100%, and specificity of 71.4% (13). This study provides preliminary clinical-assessment evidence from a selected degenerative disc disease context.

### 3.3 Disc and endplate MRI findings

Degenerative MRI findings are common in asymptomatic populations and increase with age. In a systematic review of asymptomatic individuals, disc degeneration increased from approximately 37% at 20 years of age to 96% at 80 years, and disc bulging increased from 30% to 84% over the same age range (8). In adults aged 50 years or younger, several MRI findings were more frequent in patients with low back pain than in asymptomatic controls, including disc bulge, disc extrusion, and Modic type 1 changes; high-intensity zones and annular fissures were not statistically significant in that analysis (7).

For high-intensity zones (HIZs), diagnostic-accuracy evidence showed higher specificity than sensitivity. A 2024 meta-analysis of 25 studies including 5,889 patients reported a pooled sensitivity of 0.49, specificity of 0.89, positive likelihood ratio of 4.52, negative likelihood ratio of 0.58, and area under the curve of 0.84 (14). Another meta-analysis, mainly based on discography-referenced studies, found that HIZ-positive disks were more likely to show abnormal morphology and concordant pain (15), whereas an observational imaging study reported low sensitivity and no clear correlation between HIZ and pain or disability scores (16).

Evidence for vertebral endplate signal changes mainly concerns associations with clinical low back pain populations. One systematic review reported a median prevalence of 43% in clinical populations and 6% in nonclinical populations, with positive associations in 7 of 10 studies (17). In a retrospective imaging study of 60 patients with DLBP and 60 controls, Song et al. reported lumbar stability, Modic change, and HIZ as independent imaging factors, with AUCs of 0.978, 0.747, and 0.717, respectively (18). Across MRI studies, comparator populations and reference criteria varied, including asymptomatic controls, symptomatic cohorts, and discography-referenced settings.

### 3.4 Alternative pain-source assessment

Studies addressing other pain sources have examined suspected DLBP within the broader differential diagnosis of chronic low back pain. These studies evaluated facet joint and sacroiliac joint sources alongside disc-related findings using discography, controlled blocks, and provocation-test clusters. In a cohort of 92 consecutive patients with chronic low back pain assessed with discography and facet joint blocks, 36 had positive discography, 8 had symptomatic facet joint pain, and 3 had positive findings for both sources (19). A controlled block study of 120 patients reported facet joint pain in 40% of patients (95% CI, 31%−49%), discogenic pain in 26% (95% CI, 18%−34%), and sacroiliac joint pain in 2% (20).

For sacroiliac joint pain, systematic reviews reported modest diagnostic performance for clusters of provocation tests. One review reported a positive likelihood ratio of 3.2 and a negative likelihood ratio of 0.29 for combined sacroiliac joint tests (11). A more recent diagnostic accuracy review of disc, sacroiliac joint, and facet joint pain reported positive likelihood ratios of approximately 2.4–3.2 for sacroiliac joint provocation test clusters (12). These findings indicate that assessment of other lumbar or pelvic pain sources can provide differential diagnostic information in suspected DLBP, particularly when symptoms and imaging findings overlap across potential sources.

### 3.5 Provocative discography and diagnostic blocks

Provocative discography is the most extensively described disc-targeted diagnostic procedure in this evidence base. Studies typically interpret results using familiar pain reproduction, disc morphology, control-level responses, and injection conditions such as volume and pressure (21–25). One systematic review reported false-positive rates of 9.3% per patient and 6.0% per disc, with a pooled specificity of 0.94 (21). In the same review, false-positive rates were lower among asymptomatic subjects without confounding factors and higher in a somatization subgroup (21). A prospective study of low-speed pressure-controlled discography reported an average intradiscal peak pressure of 15.1 psi (SD 11.1), with minimal pressure change in adjacent disks (25).

Diagnostic block studies evaluated pain relief or pain modification after discoblock, L2 nerve root infiltration, or selective nerve blocks. These procedures provide diagnostic information that differs from provocative discography: discography evaluates whether controlled disc stimulation reproduces familiar pain, whereas blocks evaluate whether targeted anesthesia reduces or modifies that pain (26–28). In a prospective observational study of 40 patients undergoing unilateral L2 spinal nerve root infiltration before discography, infiltration results agreed with provocative discography in 46.5% of cases (26).

## 4 Discussion

This review shows that suspected DLBP is evaluated through several complementary forms of diagnostic information. Clinical features indicate whether a disc-related diagnosis is clinically compatible, MRI identifies disc and endplate structural findings, alternative pain-source assessment places those findings within the broader differential diagnosis, and provocative discography or diagnostic blocks provide selected procedural responses. Together, these evidence areas support clinical evaluation by bringing clinical presentation, imaging, differential assessment, and procedural findings into the same diagnostic context.

### 4.1 Clinical features

Clinical features mainly help determine whether a disc-related explanation is plausible in the individual presentation. Axial low back pain, sitting or flexion intolerance, and non-radicular referred pain may support this explanation when they are prominent, but these symptoms are not specific enough to identify a disc source on their own (3, 9, 10). Their main role is to define the clinical context in which disc and endplate MRI findings, or selected procedural tests, can be interpreted more meaningfully.

Among the clinical findings reviewed, centralization provides the most consistent support for a disc source, although its diagnostic effect is moderate rather than definitive (11, 12, 29). A positive centralization response can increase suspicion of disc-related pain, whereas its absence does not exclude DLBP. Interview-based support tools may also improve clinical assessment in selected degenerative disc disease populations, but they require further validation before broader application (13). In practice, clinical features should guide the need for subsequent imaging or selected procedural evaluation, rather than serve as stand-alone diagnostic criteria.

### 4.2 Interpreting disc and endplate MRI findings

Disc degeneration and disc bulging are frequent MRI findings; when present alone, they provide limited support for attributing low back pain to a disc source (7, 8). Annular fissuring and HIZs provide more specific information related to annular disruption, although diagnostic evidence is more consistently quantified for HIZ. Given its relatively high specificity but modest sensitivity, a level-concordant positive HIZ can increase suspicion of a disc-related pain source, whereas the absence of HIZ does not exclude DLBP (14–16).

Modic changes and other endplate-related signal changes may suggest involvement of the vertebral endplate and the adjacent disc in the pain process. Systematic reviews have reported associations between these findings and low back pain, although the strength and consistency of these associations vary according to imaging definitions, study populations, and comparator groups (17, 30–32). In suspected DLBP, their main value is to strengthen an anatomically plausible explanation that the suspected disc level and adjacent endplate region may be contributing to the patient's pain, rather than to serve as independent diagnostic criteria.

Combined imaging interpretation may be more informative than isolated MRI signs in selected populations. The findings of Song et al., in which lumbar stability, Modic change, and HIZ were identified as independent imaging factors, illustrate this potential value (18). However, they do not establish MRI as a stand-alone diagnostic standard. Routine patient-level use of combined MRI patterns still requires prospective validation with standardized imaging definitions and clearly described clinical populations (18, 33).

### 4.3 Alternative pain-source assessment

Alternative pain-source assessment helps prevent common disc findings from being given excessive diagnostic weight. After clinical features and MRI suggest a possible disc source, the key question is whether another common source of low back pain better fits the patient's presentation. This step is necessary because disc, facet joint, and sacroiliac joint pain can overlap in location, and pain location alone does not reliably separate them (19, 20). Targeted examinations, sacroiliac joint provocation-test clusters, and controlled facet joint or sacroiliac joint blocks can redirect interpretation when their findings fit a non-disc source, but they should inform rather than determine the diagnosis (11, 12, 19, 20). When another source fits better, disc degeneration or other disc-related MRI findings should be interpreted with caution. When no other source is more convincing and the remaining diagnostic information supports the suspected level, a disc-related interpretation becomes more coherent.

### 4.4 Provocative discography and diagnostic blocks

Although provocative discography has often been used as a reference or confirmatory test in studies of discogenic pain, its result should not be treated as an unconditional gold standard. In suspected DLBP, its main contribution is to test whether stimulation of a clinically suspected disc reproduces the patient's familiar pain, rather than to identify painful discs from morphology alone. A concordant response can support a disc-related explanation when it occurs at the suspected level and fits the broader clinical and imaging context (23–25).

The interpretability of provocative discography depends on how the response is obtained. Injection pressure and volume, flow rate, criteria for pain concordance, control-level responses, patient selection, and psychosocial factors, including somatization-related features, can all influence whether a positive response reasonably reflects the suspected disc or a false-positive reaction (21, 23–25, 34, 35). In selected patients, brief psychosocial screening with instruments such as the Örebro Musculoskeletal Pain Screening Questionnaire or the STarT Back Screening Tool may help identify relevant psychosocial factors and prognostic risk for persistent disability (36, 37), thereby providing additional context for interpreting discography or diagnostic block responses. These considerations are therefore not merely technical details; they affect how much diagnostic weight should be assigned to procedural pain responses.

Diagnostic blocks address a different response. Discoblock, L2 spinal nerve root infiltration, and related anesthetic procedures assess whether targeted anesthesia reduces or modifies the patient's familiar pain, rather than whether pain can be reproduced by stimulation (26–28). Incomplete agreement between L2 infiltration or functional anesthetic discography and provocative discography suggests that pain reproduction and pain relief responses may be related, but they should not be interpreted as interchangeable (26, 28).

These procedures are best reserved for selected patients in whom clinical features, MRI findings, and assessment of other pain sources leave an unresolved question about the suspected level. In that setting, pain reproduction during discography or pain relief after targeted anesthesia can help clarify whether the suspected level is linked to the patient's familiar pain. Their results should therefore be interpreted as context-dependent support, not as stand-alone confirmation of DLBP.

### 4.5 Future directions

Future research should address a central unresolved task in suspected DLBP: determining whether abnormalities in a disc or adjacent endplate are meaningfully related to the patient's familiar pain. This question is unlikely to be resolved by another isolated diagnostic marker alone. Instead, it requires prospective validation of integrated diagnostic strategies that combine information from the main diagnostic domains and test whether such integration improves diagnostic agreement, identification of the clinically relevant level, and selection for further diagnostic procedures.

For this evidence to become clinically useful, future studies need more consistent definitions and transparent reporting. Key MRI findings should be defined using explicit and reproducible criteria. Studies of provocative discography or diagnostic blocks should clearly describe how pain reproduction or pain relief is assessed and interpreted (21, 23–28, 34, 35). New markers or multivariable tools should be evaluated according to whether they add reproducible and clinically useful information beyond isolated structural abnormalities, rather than by apparent performance in selected samples alone (38, 39). This would move the field toward a more reliable diagnostic approach for suspected DLBP without treating any single finding as definitive.

### 4.6 Strengths and limitations

A strength of this review is that it addresses the diagnostic problem underlying suspected DLBP: structural disc or endplate abnormalities, low back pain, and the suspected spinal level do not correspond in a simple one-to-one manner. By organizing the literature around this problem, the review clarifies how different sources of diagnostic information may alter the interpretation of common disc findings in an individual patient. Its contribution is therefore interpretive rather than algorithmic: it summarizes the diagnostic value and boundaries of current evidence without proposing a new diagnostic standard.

The limitations arise from both the review design and the underlying evidence base. This was a focused narrative review rather than a formal diagnostic test accuracy review or meta-analysis, and it was not designed to generate pooled accuracy estimates, diagnostic thresholds, or a fixed diagnostic pathway. The available studies also differ in design, patient selection, imaging definitions, reference standards, discography criteria, and block techniques, and many evaluated single evidence domains rather than integrated diagnostic assessment. Accordingly, the conclusions are best read as an interpretive synthesis of how current evidence may be weighed in suspected DLBP, rather than as definitive diagnostic criteria.

## 5 Conclusions

The diagnosis of suspected DLBP should not be based on disc degeneration or any isolated finding alone. Clinical presentation, disc and endplate MRI findings, assessment of other possible pain sources, and selected responses to provocative discography or diagnostic blocks can provide complementary diagnostic information when interpreted in relation to the patient's familiar pain and the suspected spinal level. Current evidence supports an integrated diagnostic approach that may improve diagnostic confidence in selected patients while preserving the interpretive limits of available tests.
